# The Missing Culprit: Spontaneous Postpartum Hemoperitoneum With an Intact Uterus and No Identifiable Bleeding Source During Surgical Exploration

**DOI:** 10.7759/cureus.112897

**Published:** 2026-07-18

**Authors:** Sowmiya Rajendran, Kanika Chopra, Mohammed Thaha S

**Affiliations:** 1 Department of Obstetrics and Gynaecology, Lady Hardinge Medical College, Delhi, IND; 2 College of Medicine, Madras Medical College, Chennai, IND

**Keywords:** emergency laparotomy, hemoperitoneum, hypovolemic shock, intra-abdominal bleeding, maternal morbidity, obstetric emergency, postpartum collapse, postpartum hemorrhage, spontaneous postpartum hemoperitoneum, vaginal delivery

## Abstract

Spontaneous hemoperitoneum in the postpartum period is a rare and potentially life-threatening obstetric emergency that may present with sudden hemodynamic collapse. We report the case of a 30-year-old gravida 3, para 1, living 1, abortion 1 woman at 39 weeks of gestation with a previous lower-segment cesarean section who underwent a successful vaginal birth after cesarean (VBAC) of a healthy female neonate weighing 2.95 kg. One hour after delivery, despite stable vital signs and absence of vaginal bleeding, she developed sudden circulatory collapse with abdominal distension and hypovolemic shock. Laboratory investigations revealed severe anemia with hemoglobin of 6.7 g/dL, thrombocytopenia, and metabolic acidosis with elevated lactate levels. Bedside ultrasonography demonstrated significant intraperitoneal free fluid, and diagnostic tapping confirmed hemoperitoneum. Emergency exploratory laparotomy revealed approximately 3.8 liters of hemoperitoneum with clots. The uterus was exteriorized and systematically examined; the previous cesarean scar was intact, with no evidence of uterine rupture or dehiscence. The adnexa, broad ligaments, pelvic and retroperitoneal regions, and upper abdominal viscera were carefully examined, but no definite source of bleeding could be identified. Stepwise surgical devascularization with bilateral uterine artery ligation and descending cervical artery ligation was performed as an empirical hemorrhage-control measure. The patient received massive transfusion support, recovered satisfactorily in the intensive care unit, and was discharged in stable condition. This case highlights the importance of maintaining a high index of suspicion for spontaneous hemoperitoneum in postpartum women presenting with unexplained shock, even in the absence of vaginal bleeding.

## Introduction

Spontaneous hemoperitoneum in pregnancy is defined as an intraperitoneal hemorrhage that manifests during pregnancy or postpartum in the absence of trauma, excluding bleeding associated with ectopic pregnancy, uterine rupture or cesarean section [1].

With an estimated incidence of approximately one in 25,000 pregnancies, it is an uncommon cause of postpartum shock and has been associated with rupture of uterine or utero-ovarian vessels, pelvic varicosities, endometriosis-related lesions, and vascular aneurysms. Owing to its nonspecific presentation and rapid progression to hemodynamic instability, early recognition and prompt surgical intervention are essential to reduce maternal morbidity and mortality [2].

During pregnancy, increased pelvic and uterine vascularity, together with increased venous pressure and vascular engorgement, may predispose uterine and pelvic vessels to spontaneous rupture [3,4]. Pregnancy-related hormonal and hemodynamic changes may further contribute to alterations in vascular integrity and increased susceptibility to rupture [5].

Spontaneous postpartum hemoperitoneum with an intact uterus and no identifiable bleeding source is a rare but life-threatening obstetric emergency. It is often concealed with minimal vaginal bleeding, presenting primarily as acute abdominal pain, hypovolemic shock, or sudden hemodynamic instability [6].

In this case, we report a patient who developed massive spontaneous postpartum hemoperitoneum with an intact uterus and no identifiable bleeding source during surgical exploration, presenting as sudden hemodynamic collapse following an uncomplicated vaginal delivery.

## Case presentation

A 30-year-old woman, gravida 3, para 1, living 1, abortion 1, at 39 weeks of gestation with a history of one previous lower-segment cesarean section and one spontaneous abortion managed medically, presented in early labor. There was no significant past medical or family history. Baseline investigations, including complete blood count, liver function tests, and kidney function tests, were within normal limits. She subsequently progressed to spontaneous vaginal delivery of a live female baby weighing 2.95 kg. Following delivery, her vital signs remained stable, and there was no evidence of vaginal bleeding.

Approximately one hour after delivery, she suddenly collapsed. On examination, she was conscious but appeared pale, with cold and clammy extremities. Her pulse rate was 120 beats/minute and of low volume, blood pressure was 76/30 mmHg, respiratory rate was 22 breaths/minute, and oxygen saturation was 96% on room air. The patient's shock index at presentation was 1.58 (heart rate 120 beats/min, systolic blood pressure 76 mmHg), indicating severe hemodynamic compromise. Urinary catheterization was performed for monitoring of urine output. Abdominal examination revealed marked abdominal distension. The uterus was well contracted, centrally placed, and corresponded to approximately a 20-week uterine size. Vaginal examination showed no active bleeding per vaginum and no evidence of perineal hematoma. Repeat per vaginal examination confirmed an intact cervix, vagina, and vault. Immediate resuscitation was initiated according to the protocol for management of hypovolemic shock.

Further investigations were performed. Arterial blood gas analysis demonstrated severe metabolic acidosis with elevated lactate levels of 7.85 mmol/L. Complete blood count revealed a hemoglobin level of 6.7 g/dL, representing an approximately 40% reduction from predelivery values. Platelet count was 110,000/µL, while the international normalized ratio (INR) was 1.05. The patient had no known coagulation abnormality before delivery, and the normal INR at presentation did not suggest overt coagulopathy. Liver and kidney function tests remained within normal limits. Bedside ultrasonography of the abdomen demonstrated significant free fluid with internal echoes suggestive of hemoperitoneum and a suspicious defect in the lower uterine segment. Diagnostic abdominal tapping subsequently confirmed the presence of blood within the peritoneal cavity. With this, a provisional diagnosis of spontaneous postpartum hemoperitoneum causing hypovolemic shock was made, and emergency exploratory laparotomy was undertaken without delay. The patient's vital signs and laboratory investigations at presentation are summarized in Table 1.

**Table 1:** Clinical characteristics, vital signs, and laboratory investigations at presentation.

Parameter	Result	Reference range
General appearance	Pale, conscious, cold, and clammy extremities	-
Pulse rate	120 beats/min	60-100 beats/min
Blood pressure	76/30 mmHg	90-120/60–80 mmHg
Respiratory rate	22 breaths/min	12-20 breaths/min
Oxygen saturation (SpO₂)	96% (room air)	≥95%
Shock index	1.58	<0.7
Arterial blood gas	Severe metabolic acidosis	pH 7.35-7.45; HCO₃⁻ 22-26 mmol/L
Serum lactate	7.85 mmol/L	0.5-2.0 mmol/L
Hemoglobin	6.7 g/dL	12.0-16.0 g/dL
Platelet count	110 × 10³/µL	150-450 × 10³/µL
International normalized ratio (INR)	1.05	0.8-1.2
Liver function tests	Within normal limits	Within normal limits
Kidney function tests	Within normal limits	Within normal limits

**Figure 1:**
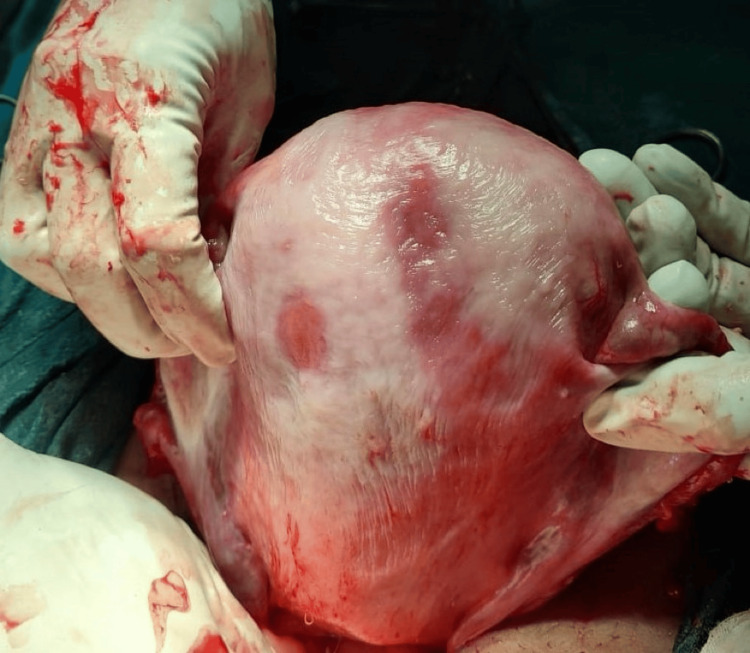
Intraoperative photograph demonstrating an intact posterior uterine wall with no evidence of uterine rupture or active pelvic bleeding during exploration.

The patient was immediately taken up for emergency exploratory laparotomy under general anesthesia. Intraoperatively, approximately 3.8 liters of hemoperitoneum with clots were evacuated. The uterus was exteriorized and systematically examined. Although ultrasonography had raised suspicion of a lower uterine segment defect, surgical exploration demonstrated an intact previous cesarean scar with no evidence of uterine rupture or dehiscence. The posterior wall of the uterus was also intact, with no evidence of uterine rupture or active pelvic bleeding (Figure 1). The uterus, bilateral adnexa, and broad ligaments appeared normal without evidence of active bleeding. The parametria, pelvic vasculature, infundibulopelvic ligaments, and retroperitoneal regions were systematically examined, with no active bleeding or retroperitoneal hematoma identified.

**Figure 2:**
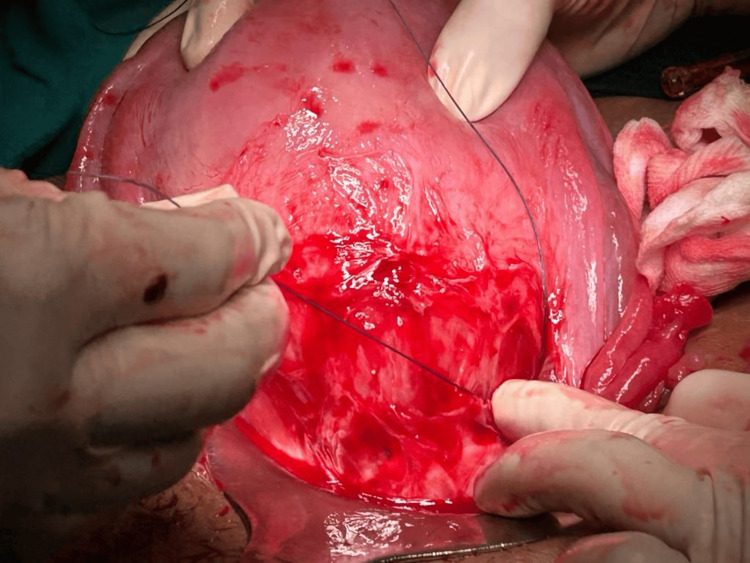
Intraoperative photograph showing an intact uterus with an intact previous lower-segment cesarean scar during exploratory laparotomy. Bilateral uterine artery ligation was performed as part of stepwise surgical devascularization for hemorrhage control.

Given the life-threatening hemoperitoneum and the possibility of an occult uterine or pelvic vascular source not apparent during exploration, stepwise surgical devascularization was undertaken as an empirical hemorrhage-control measure. Bilateral uterine artery ligation and descending cervical artery ligation were performed to reduce pelvic arterial inflow and pulse pressure, thereby facilitating hemostasis at any occult or diffuse bleeding site (Figure 2). A repeat vaginal examination again confirmed an intact cervix, vagina, and vault.

As no obvious pelvic source of bleeding was identified, the abdominal incision was extended with surgical assistance to facilitate upper abdominal exploration. The spleen, liver, lesser sac, stomach, and bowel were thoroughly examined; the mesentery and diaphragmatic regions were also inspected; however, no definitive bleeding source could be identified. Although an occult microscopic venous or arterial rupture could not be completely excluded, meticulous inspection of the previous cesarean scar, anterior and posterior uterine walls, bilateral adnexa, broad ligaments, parametria, infundibulopelvic ligaments, retroperitoneum, pelvic vasculature, mesentery, diaphragmatic regions, and upper abdominal organs failed to demonstrate any active bleeding point. An intra-abdominal drain was placed in the pouch of Douglas, and the abdomen was closed.

Postoperatively, the patient received four units of packed red blood cells, four units of fresh frozen plasma, and four units of platelets. She was transferred to the intensive care unit and managed with elective mechanical ventilation. Her clinical condition gradually improved, and she was successfully extubated on postoperative day 2. Serial laboratory investigations showed progressive hematological recovery. Before discharge, hemoglobin had improved to 9.9 g/dL, platelet count was 1.2 lakh/µL, INR remained 1.05, and liver and kidney function tests were normal. She remained hemodynamically stable and was discharged in satisfactory condition on postoperative day 8.

## Discussion

Spontaneous hemoperitoneum in pregnancy (SHiP) is characterized by spontaneous intraperitoneal bleeding without prior trauma during pregnancy and up to 42 days after delivery, necessitating surgical intervention or embolization, while excluding ectopic pregnancy, uterine rupture, and cesarean section-associated bleeding. It often presents with concealed intra-abdominal bleeding and can rapidly progress to hemodynamic instability, resulting in significant maternal and perinatal morbidity and mortality [1].

Because vaginal bleeding may be minimal or absent, diagnosis can be challenging. Patients commonly present with abdominal distension, hypovolemic shock, or sudden hemodynamic collapse, making prompt recognition and surgical intervention essential [2,6]. In our case, the patient developed sudden hemodynamic collapse one hour after a successful vaginal birth after cesarean (VBAC), despite the absence of vaginal bleeding and the presence of a well-contracted uterus.

The differential diagnosis of spontaneous postpartum hemoperitoneum includes rupture of uterine or utero-ovarian vessels, pelvic varicosities, vascular pseudoaneurysms, endometriosis-related lesions, visceral injury, and coagulation disorders. In the present case, extensive pelvic and upper abdominal exploration did not reveal any of these identifiable structural sources.

Coagulopathy and disseminated intravascular coagulation were considered among the potential causes or contributors to the hemorrhage. However, the patient had no known coagulation abnormality before delivery, and the normal INR of 1.05 at presentation did not support overt coagulopathy as the primary cause. A complete coagulation profile, including fibrinogen and D-dimer levels, was unavailable due to the need for immediate life-saving surgical intervention.

Boztosun et al. reported a case of SHiP in the early postpartum period in which no identifiable cause of bleeding could be found, similar to our case. They postulated bleeding from endometriotic foci, adhesion bands, or ectopic decidual reaction as possible mechanisms [7]. Similar to the case reported by Boztosun et al., extensive intraoperative exploration in our patient failed to identify a definitive source of bleeding despite the presence of massive hemoperitoneum. Cases of spontaneous postpartum hemoperitoneum with an intact uterus and no demonstrable bleeding source despite extensive surgical exploration are exceedingly rare, which adds to the clinical significance of the present report.

In cases of severe or intractable obstetric hemorrhage, stepwise surgical devascularization, including bilateral uterine artery ligation, provides a conservative, uterine-sparing approach that may help avoid hysterectomy and preserve fertility [8]. By reducing uterine and pelvic arterial inflow and pulse pressure, devascularization may facilitate hemostasis at occult or diffuse bleeding sites that are not readily identifiable during surgical exploration [9]. Ligation of the descending branches of the uterine arteries can further reduce the vascular supply to the lower uterine segment, cervix, and upper vagina and has been described as an additional surgical measure for controlling persistent bleeding from these regions [10].

In our case, given the life-threatening hemoperitoneum and the possibility of an occult uterine or pelvic vascular source despite the absence of an identifiable active bleeding point, bilateral uterine artery ligation and descending cervical artery ligation were performed as an empirical, uterus-preserving hemorrhage-control measure. The uterus possesses an extensive collateral arterial network with both ipsilateral and contralateral anastomoses, which may help maintain uterine perfusion following surgical devascularization [11].

Unlike many previously reported cases in which a ruptured uterine vessel, pelvic varicosity, aneurysm, or endometriotic lesion was identified, extensive pelvic and upper abdominal exploration in our patient failed to identify a definite source of bleeding. Such cases of postpartum SHiP with no identifiable bleeding source remain exceptionally uncommon and represent the principal learning point of this report.

## Conclusions

Spontaneous hemoperitoneum should be considered in any postpartum woman presenting with unexplained hypovolemic shock, abdominal distension, and a significant fall in hemoglobin despite minimal or absent vaginal bleeding. A bleeding source may remain unidentifiable despite meticulous surgical exploration. Early recognition, aggressive resuscitation, timely laparotomy, and multidisciplinary management are essential for improving maternal outcomes.
